# Sex-Specific Risk of Cardiovascular Disease in Autoimmune Addison Disease—A Population-Based Cohort Study

**DOI:** 10.1210/jc.2018-02298

**Published:** 2019-01-03

**Authors:** Jakob Skov, Anders Sundström, Jonas F Ludvigsson, Olle Kämpe, Sophie Bensing

**Affiliations:** 1 Department of Molecular Medicine and Surgery, Karolinska Institutet, Stockholm, Sweden; 2 Centre for Pharmacoepidemiology, Department of Medicine (Solna), Karolinska Institutet, Stockholm, Sweden; 3 Department of Medical Epidemiology and Biostatistics, Karolinska Institutet, Stockholm, Sweden; 4 Center for Molecular Medicine, Department of Medicine (Solna), Karolinska Institutet, Stockholm, Sweden; 5 Department of Endocrinology, Metabolism and Diabetes, Karolinska University Hospital, Stockholm, Sweden; 6 K.G. Jebsen Center for Autoimmune Diseases, University of Bergen, Bergen, Norway

## Abstract

**Context:**

Little is known of cardiovascular disease (CVD) in autoimmune Addison disease (AAD). Inadequate glucocorticoid replacement might potentially increase CVD risk.

**Objective:**

To examine CVD in AAD in subgroups of ischemic heart disease (IHD) and cerebrovascular disease (CeVD) and investigate the effects of glucocorticoid and mineralocorticoid dosing.

**Design, Setting, and Patients:**

In this cohort-control study, we used Swedish health registries from 1964 to 2013 to identify 1500 subjects with AAD and 13,758 matched controls. Incident CVD was analyzed from 2006 to 2013. Adjusted hazard ratios (aHRs) were calculated using Cox proportional hazard models. Glucocorticoid and mineralocorticoid doses were stratified to examine dose-related risks.

**Results:**

During 8807 person-years (PY), 94 events of first CVD (10.7/1000 PY) in patients with AAD occurred compared with 563 events during 80,163 PY (7.0/1000 PY) in controls. IHD was significantly more common in women (aHR, 2.15; 95% CI, 1.49 to 3.10) but not men (aHR, 1.16; 95% CI, 0.75 to 1.78) with AAD compared with controls. No increase in CeVD risk was detected (aHR, 0.88; 95% CI, 0.56 to 1.37, women; aHR, 0.88; 95% CI 0.53 to 1.50, men). CVD was associated with greater glucocorticoid and mineralocorticoid replacement doses in women but not men.

**Conclusion:**

The risk of IHD but not CeVD is increased in AAD, especially in women. The risk of CVD independently correlated with greater glucocorticoid and mineralocorticoid replacement doses in women. Our data suggest that close monitoring and early treatment of risk factors for CVD, among women in particular, might be warranted.

## Teaser

CVD risk in patients with Addison disease is primarily the result of coronary events in women. The glucocorticoid and mineralocorticoid doses correlated with the risk of CVD.

Autoimmune Addison disease (AAD) is the predominant cause of primary adrenal insufficiency in the world. Prevalence estimates have ranged from 9 to 22 per 100,000 in white populations (1). The loss of adrenocortical function in AAD is usually substituted with oral hydrocortisone two to three times daily, with doses averaging 28 mg/d in Sweden (2). Mineralocorticoid replacement has been used by ~90% of patients, with oral fludrocortisone 0.1 mg once daily as the standard treatment (2).

The mortality rates have been approximately two times greater in those with AAD than in matched control populations, primarily owing to cardiovascular disease (CVD) (3–5). In the general population, sudden cardiac death will be preceded by some form of cardiac disease in approximately two thirds of patients (6). This ratio might well be lower in those with AAD, given that these patients cannot respond to stress with glucocorticoid release and have a blunted release of adrenal catecholamines (7). To the best of our knowledge, no studies have been performed on incident CVD in patients with AAD or other forms of adrenal insufficiency. Thus, it is unknown whether the CVD mortality observed is purely a reflection of increased prevalence or whether an increased case-fatality rate is a contributing factor.

Studies comparing cardiovascular risk factors in cohorts of patients with adrenal insufficiency of predominantly autoimmune etiology and adequately matched controls, are rare. To the best of our knowledge, they have all evaluated multiple risk factors (measured or treated for), such as dyslipidemia, body mass index, abdominal adiposity, glucose metabolism, or hypertension. Although the results generally indicate unfavorable metabolic profiles in patients with AAD, they do so inconsistently (2, 8–11).

The detrimental effects of prolonged exposure to high doses of glucocorticoids have been well documented (12). The glucocorticoid replacement doses used in adrenal insufficiency are greater than the physiologic release of 5.4 to 6.1 mg/m^2^/d (13, 14). This has been suggested as a possible cause of the excess CVD in this patient group. Epidemiologic data on AAD corroborating this theory are sparse, with only one study reporting an increased prevalence of hypertension in subjects receiving higher replacement doses (2). Studies of secondary adrenal insufficiency, however, have reported adverse metabolic profiles and increased mortality for patients using higher glucocorticoid replacement doses (15, 16). Despite fundamental differences in disease etiology and comorbidities, data on secondary adrenal failure have been routinely cited in the treatment recommendations for AAD owing to a lack of better evidence (1, 17). The negative cardiovascular effects of aldosteronism have been well documented (18); however, studies on the long-term effects of mineralocorticoid replacement regimens are, to the best of our knowledge, nonexistent.

The aim of the present study was to investigate the incidence of cardiovascular morbidity, mortality, and case-fatality rate in patients with AAD. Furthermore, we explored CVD risk in relation to the glucocorticoid and mineralocorticoid replacement doses. Thus, we performed a population-based cohort-control study, collecting the diagnostic records and prescription data for an unbiased cohort representing most Swedish patients with AAD.

## Methods

The ethics review board of Stockholm, Sweden approved the present study (approval no. DNR 2006/026/3). The requirement for informed consent was waived by the ethics committee.

### Registries

The unique Swedish personal identification numbers enabled us to link the following population-based registers: Swedish National Patient Register, Swedish Prescribed Drug Register, and Cause of Death Register.

#### Swedish National Patient Register

The Swedish National Patient Register (NPR) contains inpatient information dating back to 1964, with nationwide coverage since 1987. It includes hospital admission dates, discharge dates, and discharge codes classified according to the International Statistical Classification of Diseases and Related Health Problems (ICD). As of 2001 data on hospital-based outpatient care have also been included. The diagnostic accuracy of discharge codes is generally very good (19).

#### Swedish Prescribed Drug Register

The Swedish Prescribed Drug Register (PDR) collects data on all prescription drugs dispensed in Sweden from July 2005 onward. It includes information on the dispensed quantity, dosage, expenditure, reimbursement, and drug type classified according to the Anatomical Therapeutical Chemical (ATC) classification system.

#### Cause of death register

The cause of death register contains data since 1961 on all deceased persons who at the time of death were registered in Sweden in the year they died. The recorded variables include the underlying cause of death, multiple causes of death, nature of injuries associated with death, and the basis for the statement of the cause of death. The diagnoses are coded according to the ICD. Coverage is >99.5% (20).

### Participants

We identified all patients with a diagnosis of AAD in the NPR and subsequently excluded all patients with ICD codes suggesting other causes of adrenal failure (21) at their first exclusion diagnosis. The PDR was used to further validate a diagnosis of AAD by requiring multiple (≥2) fillings of prescriptions for either hydrocortisone or cortisone acetate (ATC H02AB09/H02AB10) and multiple (≥2) fillings of prescriptions for fludrocortisone (ATC H02AA02). Using the population register, we randomly identified 10 controls for each AAD subject, individually matched for birth year, sex, and county of residence. The controls were also matched by the date of the first-ever ICD diagnosis of AAD in their counterpart.

### Outcome measures and covariates

**Figure 1.**
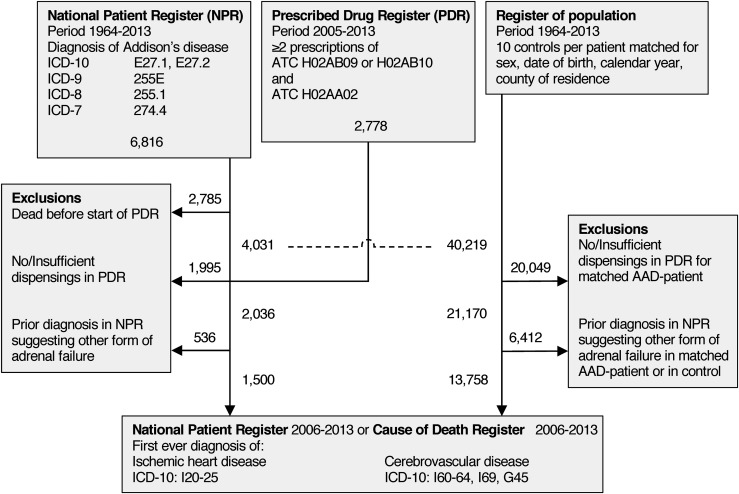
Flowchart of study participants. Dashed line indicates point of matching patients with AAD to controls.

CVD was defined as nonfatal or fatal events of CVD with a corresponding main diagnosis according to the 10th revision of the ICD (ICD-10; Fig. 1). Fatal CVD was defined as death from CVD within 30 days of a CVD event. The outcome was subdivided into ischemic heart disease (IHD) and cerebrovascular disease (CeVD). Hypertension was defined by multiple (≥2) fillings of prescriptions for antihypertensive agents. Dyslipidemia was defined by multiple (≥2) fillings of prescriptions for statins or other lipid-modifying agents. Type 1 diabetes was defined by the corresponding ICD-10 codes. Among the subjects with ICD-10 codes for both type 1 and type 2 diabetes, the last recorded diagnosis before the start of follow-up was used to determine the type of diabetes. All other subjects with a diagnostic record indicating diabetes or with multiple fillings of prescriptions for antidiabetic agents were considered to have type 2 diabetes. Hashimoto thyroiditis was defined according to the ICD codes and prescription patterns, as reported previously (22). Chronic obstructive pulmonary disease (COPD), defined by the ICD codes, was used as a proxy for heavy smoking. The diagnoses and events were identified in the NPR and the cause of death register and the prescription patterns in the PDR, as detailed in the online repository (21).

### Statistical analysis

The number of person-years (PY) at risk was calculated separately for those with AAD and controls as the sum of years during follow-up. The start of follow-up was defined as the time of the second filling of glucocorticoids, mineralocorticoids, or 1 January 2006, whichever occurred last. By default, the patients with AAD and controls who had died before 1 January 2006 were excluded from follow-up period. Moreover, the controls alive at the start of follow-up were excluded if their matched patient with AAD had died. The end of follow-up occurred at the time of death, entry of an exclusion diagnosis, or the end of the study period (31 December 2013), whichever occurred first.

The mean daily doses of hydrocortisone and fludrocortisone were estimated by dividing the total dispensed quantities during the study by time under study (in days) for each patient. Cortisone acetate doses were translated into hydrocortisone-equivalent doses (1 mg of cortisone acetate/1.25 = 1 mg of hydrocortisone). The hydrocortisone doses were normally distributed, and fludrocortisone doses had a bimodal distribution (21). Hence, tertiles of low, intermediate, and high doses of hydrocortisone and halves of low and high doses of fludrocortisone were used for the evaluation of the dose-related risks.

Multiple Cox regression models were used to estimate the hazard ratios (HRs) with 95% CIs for CVD, IHD, and CeVD to compare the subjects with AAD to population controls. The models were internally matched for sex, age, and county of residence by stratification. Adjustments were made for the presence of diabetes and COPD. When exploring the correlations between replacement therapy and outcomes, dummy variables of hydrocortisone doses in tertiles and fludrocortisone doses in halves were added to the Cox models. To examine the overall replacement regimens, a dummy variable with the subjects assigned to one of six strata according to the hydrocortisone tertile and fludrocortisone half were analyzed in a separate Cox model.

The 30-day case fatality rates for IHD and CeVD, defined as the proportion of events leading to death (from the same cause) within 30 days, were calculated with the 95% CIs around the difference in proportion. The level of significance was set to *P* < 0.05. The statistical analyses were performed using Stata, version 11.2 (StataCorp, College Station, TX).

## Results

A total of 1500 patients with AAD and 13,785 matched controls were identified by linking the national health and population registries. The ascertainment process is outlined in Fig. 1. The baseline characteristics of the matched cohort are listed in Table 1. During 8807 PY for the patients with AAD, 94 events of the first CVD (10.7/1000 PY) occurred compared with 563 events during 80,163 PY (7.0/1000 PY) in the matched controls. This corresponded to a HR of 1.52 (95% CI, 1.21 to 1.89). Adjustment for diabetes and COPD returned an adjusted HR (aHR) of 1.20 (95% CI, 0.95 to 1.51) for incident CVD. Subjects with AAD were more likely than controls to develop IHD (aHR, 1.61; 95% CI, 1.22 to 2.12). In contrast, CeVD did not appear to be more common in those with AAD (aHR, 0.88; 95% CI, 0.63 to 1.23; Table 2).

**Table 1.** Baseline Characteristics of Patients and Controls

Characteristic	AAD			Controls		
	Total	Women	Men	Total	Women	Men
Individuals, n (%)	1500	818 (54.5)	682 (45.5)	13 758	7487 (54.4)	6271 (45.6)
Age, y (median; IQR)						
At first diagnosis	37 (26–51)	42 (29–54)	33 (25–49)	36 (25–49)	40 (29–53)	32 (22–44)
At start of follow-up	50 (37–63)	54 (40–67)	47 (33–59)	49 (36–62)	52 (39–65)	45 (33–57)
Decade of study entry,^*a*^ n (%)						
1960	15 (1.0)	9 (1.1)	6 (0.9)	127 (0.9)	78 (1.0)	49 (0.8)
1970	154 (10.3)	79 (9.7)	75 (11.0)	1221 (8.9)	625 (8.3)	596 (9.5)
1980	318 (21.2)	167 (20.4)	151 (22.1)	2787 (20.3)	1442 (19.3)	1345 (21.4)
1990	311 (20.7)	169 (20.7)	142 (20.8)	2906 (21.1)	1578 (21.1)	1328 (21.2)
2000	514 (34.3)	287 (35.1)	227 (33.3)	4904 (35.6)	2727 (36.4)	2177 (34.7)
2010	188 (12.5)	107 (13.1)	81 (11.9)	1813 (13.2)	1037 (13.9)	776 (12.4)
Hypertension, n (%)	353 (23.5)	134 (16.4)	219 (32.1)	2 815 (20.5)	1770 (23.6)	1045 (16.7)
Dyslipidemia, n (%)	189 (12.6)	113 (13.8)	76 (11.1)	1 219 (8.9)	683 (9.1)	536 (8.5)
Diabetes, n (%)	266 (17.7)	140 (17.1)	126 (18.5)	649 (4.7)	362 (4.8)	287 (4.6)
Type 1	197 (13.1)	98 (12.0)	99 (14.5)	141 (1.0)	77 (1.0)	64 (1.0)
Type 2	69 (4.6)	42 (5.1)	27 (4.0)	508 (3.7)	285 (3.8)	223 (3.6)
Hashimoto thyroiditis	542 (36.1)	364 (44.5)	178 (26.1)	501 (3.6)	438 (5.9)	63 (1.0)
COPD, n (%)	18 (1.2)	12 (1.5)	6 (0.9)	113 (0.8)	72 (1.0)	41 (0.7)
Previous CVD,^*b*^ n (%)						
Before first diagnosis	31 (2.1)	13 (1.6)	18 (2.6)	356 (2.6)	176 (2.4)	180 (2.9)
Before start of follow-up	100 (6.7)	53 (6.5)	47 (6.9)	1079 (7.8)	567 (7.6)	512 (8.2)
Follow-up, y (median; IQR)	7.5 (3.7–8.0)	7.1 (3.3–8.0)	7.8 (4.6–8.0)	7.4 (3.7–8.0)	6.9 (3.2–8.0)	7.7 (4.4–8.0)

Abbreviation: IQR, interquartile range.

^a^ Equal to year of diagnosis for the patients; follow-up for all analyses started at the filling of the second prescription of replacement therapy or 1 January 2006, whichever occurred last.

^b^ Including CeVD.

**Table 2.** Unadjusted and aHRs for Cardiovascular Events in Subjects With AAD vs Matched Controls, Stratified by Sex

Outcome	AAD (n = 1500)		Controls (n = 13,758)		Crude HR	95% CI	*P* Value	aHR^*a*^	95% CI	*P* Value
	Events, n	Events/1000 PY	Events, n	Events/1000 PY						
CVD^*b*^^,^^*c*^										
All	94	10.7	563	7.0	1.52	1.21-1.89	0.003^*d*^	1.20	0.95-1.51	0.13
Male	40	10.3	270	7.6	1.35	0.97-1.88	0.08	1.05	0.74-1.50	0.79
Female	54	12.7	293	7.6	1.68	1.25-2.25	<0.001^*d*^	1.35	0.98-1.85	0.06
IHD										
All	71	8.1	338	4.2	1,92	1.48-2.47	<0.001^*d*^	1.61	1.22-2.12	0.001^*d*^
Male	28	7.0	181	5.0	1.41	0.95-2.10	0.09	1.16	0.75-1.78	0.50
Female	43	9.8	157	3.9	2.50	1.79-3.51	<0.001^*d*^	2.15	1.49-3.10	<0.0001^*d*^
CeVD										
All	44	5.0	343	4.3	1.17	0.85-1.60	0.33	0.88	0.63-1.23	0.46
Male	19	4.7	144	3.9	1.21	0.75-1.95	0.44	0.88	0.53-1.50	0.63
Female	25	5.6	199	4.9	1.14	0.75-1.73	0.53	0.88	0.56-1.37	0.57

^a^ Adjusted for diabetes and COPD.

^b^ CVD included IHD and CeVD.

^c^ Number of events might not equal the sum of IHD and CeVD because both can occur in the same individual.

^d^ Statistically significant.

In a separate analysis of individuals with a first-ever record of AAD after 1 January 2006, with the PDR in operation, CVD events were rare. The point estimates for CVD, IHD, and CeVD and dose effects were in line with the presented results but with broad CIs, precluding further analysis (data not presented).

### Sex-related risks

The women were more prone to develop IHD (aHR, 2.15; 95% CI, 1.49 to 3.10) than were the men (aHR, 1.16; 95% CI, 0.75 to 1.78; *P* for interaction = 0.036). For CeVD, no statistically significant sex effects were observed (Table 2).

### Dose-related risks

**Figure 2.**
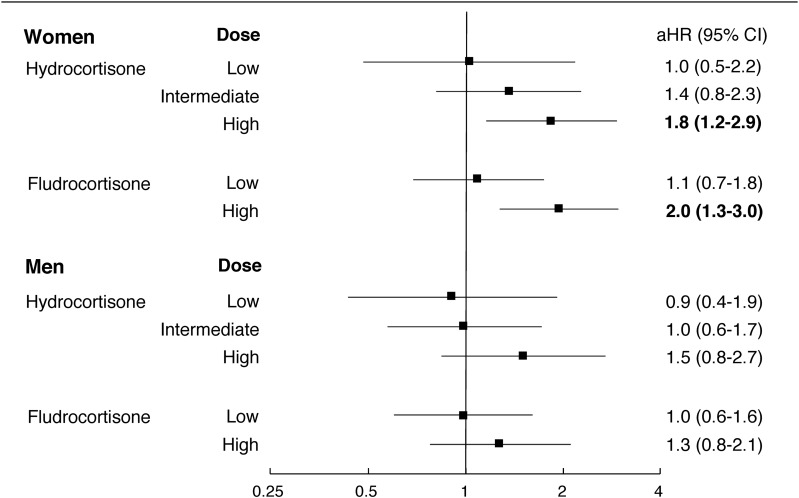
aHRs for CVD in subjects with AAD vs matched controls according to sex and hydrocortisone/fludrocortisone dosing, adjusted for diabetes and COPD in all models and tertiles of hydrocortisone dosing or halves of fludrocortisone dosing, as appropriate.

**Figure 3.**
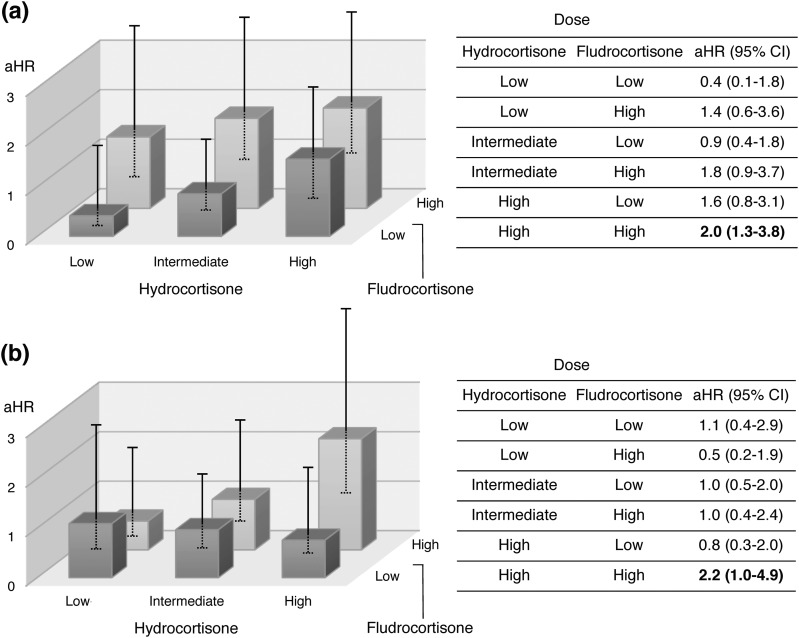
aHRs for CVD in (a) women and (b) men with AAD according to combinations of tertiles of hydrocortisone doses and halves of fludrocortisone doses, adjusted for diabetes and COPD. Error bars indicate 95% CIs.

The mean dispensed doses during the follow-up period were 29.6 ± 0.6 mg/d for hydrocortisone equivalents and 0.09 ± 0.04 mg/d for fludrocortisone. The doses were slightly higher for the men than for the women (Table 3). The associations between the glucocorticoid and mineralocorticoid replacement doses and HRs for CVD are displayed in Fig. 2. An incremental increase in the aHR, with wide CIs, was observed with higher doses of both hydrocortisone and fludrocortisone. This effect was present in both men and women. However, only high dose use in women produced statistically significant increases in the risk of CVD. Figure 3 displays aHRs for CVD in the strata of glucocorticoid–mineralocorticoid combinations. Again, a dose-risk relationship was most apparent in women. However, HRs were significantly elevated among the high dose users (both drugs), irrespective of sex.

**Table 3.** Replacement Doses of Hydrocortisone in Tertiles and Fludrocortisone in Halves

Drug	Replacement Dose
Hydrocortisone,^*a*^ mg/d	
Low	
Women	18.4 (2.7–24.0)
Men	20.1 (4.6–27.6)
Intermediate	
Women	28.1 (24.0–30.9)
Men	30.9 (27.7–34.6)
High	
Women	37.6 (30.9–73.4)
Men	42.9 (34.6–131)
Fludrocortisone, mg/d	
Low	
Women	0.06 (0.004–0.091)
Men	0.07 (0.006–0.097)
High	
Women	0.11 (0.091–0.91)
Men	0.12 (0.097–0.63)

^a^ Cortisone acetate doses divided by 1.25 for hydrocortisone-equivalent doses.

### Case-fatality rate at 30 days

In those with AAD, 29 of 71 IHD events (40.8%) were fatal compared with 96 of 338 events (28.4%) in the controls (*P* = 0.04). For those with CeVD, no discernible difference was found in the 30-day case-fatality rate, with 6 of 44 events (13.6%) fatal in the AAD cohort and 45 of 343 events (13.1%) fatal among controls (Table 4).

**Table 4.** 30-d Case-Fatality Rate in IHD and CeVD

Outcome	AAD			Controls			Difference in Proportion	95% CI	*P* Value
	Fatal Events, n	Nonfatal Events, n	Proportion	Fatal Events, n	Nonfatal Events, n	Proportion			
IHD	29	42	0.408	96	242	0.284	0.124	0.0004 to 0.248	0.04
CeVD	6	38	0.136	45	298	0.131	0.005	−0.102 to 0.113	0.92

## Discussion

To the best of our knowledge, the present study is the first to examine the CVD patterns and potential effects of replacement therapy for patients with AAD. We found that patients with AAD had an increased risk of CVD compared with matched controls and that this risk primarily resulted from IHD in the female patients. We also found that the risk of CVD correlated with replacement therapy doses for both hydrocortisone and fludrocortisone. This pattern was most pronounced in women. Moreover, we have demonstrated that subjects with AAD were more likely than were the controls to die if they experienced a coronary event.

Previous studies of AAD have focused on exploring the overall causes of death. Thus, the cardiovascular definitions were in accordance with the chapters in the 9th and 10th revisions of ICD and often included both cardiopulmonary diseases without arteriosclerotic etiology and diseases of the venous circulation (3–5). We are only aware of one earlier report that examined IHD and CeVD specifically, which reported that 55% and 19% of the total CVD deaths in the patients with AAD were accountable to these conditions (3). Our findings have built on these data by demonstrating increased morbidity using the conventional definitions of CVD.

The patterns of CVD in those with AAD differ from what has been reported for other autoimmune diseases. In rheumatic disorders, an increased risk of CVD has been observed, with only modest differences in the risk of CeVD vs IHD (23). In patients with type 1 diabetes, the risk of IHD will be more pronounced, but an increased risk of CeVD is nevertheless apparent (24). In patients with AAD, however, we found no evidence of CeVD being more common than in controls; thus, IHD alone, primarily in women, resulted in the excess in CVD risk. Heavy smoking and diabetes are slightly stronger risk factors for CVD in women than in men. In contrast, hypertension, dyslipidemia, and obesity reveal no convincing sex differences (25). In our cohort, dyslipidemia was more common in patients with AAD than in the controls; however, the difference was modest. The presence of hypertension is unlikely to explain our findings. It was more common in men but less common in women with AAD compared with the controls. Moreover, CeVD, with hypertension the single most important risk factor, was less common in those with AAD than in the controls. We did not have information on body weight. However, a previous study examining a large cohort of Swedish patients with AAD, with data partly overlapping with our cohort, reported a lower prevalence of a body mass index of ≥25 kg/m^2^ in subjects with AAD than in the matched controls (2). The reported data on tobacco use by patients with AAD are scarce. However, a recent study of a sample of Swedish patients reported a smoking prevalence of only 4% (8). In the present study, COPD, a proxy for heavy smoking, was uncommon in both patients and controls. Thus, the classic risk factors for CVD do not offer a comprehensive explanation of our findings, suggesting that other causes must be considered.

In autoimmune rheumatic diseases, systemic inflammation is believed to contribute to accelerated atherosclerosis through proatherogenic and prothrombotic processes (26). Similar mechanisms have been suggested in organ-specific autoimmunity, with evidence of inflammatory biomarkers linked to CVD persisting after end organ destruction in both AAD and Hashimoto thyroiditis (8, 27). Regardless of the disease mechanism, autoimmune comorbidities most likely explain some of the CVD risks in those with AAD. This is most evident for the combination of AAD and type 1 diabetes, with CVD mortality much greater than for those with diabetes alone (28). This combination appears to account for most of the excess CVD observed in the male patients with AAD, with the elevated CVD risk nullified after adjustment for diabetes (type 1 or type 2) and COPD. Although autoimmune diabetes is slightly more common in men than in women with AAD, most other autoimmune diseases reported in conjunction with AAD have a female preponderance. Hypothyroidism, affecting nearly one half of all female patients in the present study, and premature ovarian failure, reported by ~5% of women with AAD (2, 29), have both been linked to an increased risk of CVD (30). Premature ovarian failure has been linked to an increased risk of IHD but has little effect on the risk of CeVD (31), analogous to the disease pattern we observed. Women with AAD also lack adrenal androgens such as dehydroepiandrosterone and testosterone. Several randomized, placebo-controlled trials have evaluated the effects of dehydroepiandrosterone substitution in those with AAD for periods of 3 to 12 months. Cardiovascular endpoints have not been examined; however, some studies have included markers of cardiovascular risk, such as lipid profiles or endothelial function (32–36). Overall, treatment does not appear to improve the risk factors for CVD, with reduced high-density lipoprotein levels the only consistent finding (32, 35, 36).

The potential reasons for the high incidence of IHD in women with AAD can be found from other settings. Studies of glucocorticoid use for anti-inflammatory purposes have reported a dose-related increase in risk of IHD and, only to a lesser extent, of CeVD (37, 38). Glucocorticoid replacement in AAD exceeds the cortisol release in healthy individuals. Thus, with serum cortisol levels possibly lower in women than in men (39), oversubstitution could be of greater concern in women. Nevertheless, we observed an incremental increase in the HRs for CVD with increasing hydrocortisone doses in both women and men.

The optimal mineralocorticoid replacement in AAD is largely unknown. The glucocorticoid dose and possibly individual variations determine the dose needed for homeostasis. Fludrocortisone doses of 0.05 to 0.2 μg/d, titrated by assessing clinical signs and symptoms such as edema, salt craving, postural hypotension, electrolyte levels, and blood pressure, have usually been recommended (17, 40, 41). Still, a large minority of patients with AAD have reported symptoms consistent with mineralocorticoid deficiency, and fludrocortisone replacement has often been in the lower range of the recommended doses (42, 43). The mean dispensed fludrocortisone doses were modest in our study. Hence, the correlation between the mineralocorticoid replacement doses and the risk of IHD in women was surprising. The simplest explanation for this observation would be that the doses of mineralocorticoid and glucocorticoid correlate and that the connection was confounded by glucocorticoid use. However, the results from our stratified analysis contradicted this explanation. Again, sex differences in hormone regulation, with lower levels of aldosterone in premenopausal women than in men and, therefore, a greater risk of overtreatment, might account for our findings (44, 45).

In patients with AAD, missed glucocorticoid doses will usually lead to acute symptoms, and doses of half tablets are often used, potentially resulting in an increased waste of the drugs. Some degree of drug hoarding is, therefore, likely; however, the mean dispensed dose of 29.6 mg/d in our cohort was only 1.5 mg/d greater than the average self-reported dose of 28.1 mg/d in the Swedish Addison Registry (2), suggesting good accuracy of the dose estimates at a group level.

In our study, IHD events were more often fatal in the AAD population than among the controls. In theory, the greater case-fatality rate might have resulted from a different pathologic etiology, resulting in more severe disease in those with AAD than in controls. However, a more likely explanation would perhaps be that adequate glucocorticoid therapy will be withheld at some point for patients with AAD during acute coronary events or during the convalescent phase, resulting in increased lethality. We did not have access to the patients’ medical records and could, therefore, not confirm this hypothesis.

Theoretically, reduced glucocorticoid dosing might increase the risk of an adrenal crisis and death from other causes; however, observational data have not supported this hypothesis (46). In our study, the lower risk of CVD observed in the low-dose tertile was not offset by an increase in overall mortality. The dose relationships with regard to overall mortality were similar to what we have reported for CVD (unpublished data).

Adrenal failure is a heterogenic condition with many potential mechanisms of disease. Primary adrenal insufficiency is most commonly caused by AAD; however, other causes include monogenic, malignant, and infectious diseases. Secondary adrenal failure due to pituitary disease and iatrogenic failure due to surgery or long-term glucocorticoid treatment are also common causes of adrenal malfunction. The different forms are easily confused and sometimes ICD-coded as AAD. This has resulted in considerable miscoding. A Norwegian study that used clinical records to validate AAD found the specificity for the ICD codes to be 60% to 66% (5). Two Swedish registry-based studies of AAD that excluded individuals with ICD codes suggesting other causes of adrenal insufficiency or lacking prescriptions for fludrocortisone and either hydrocortisone or cortisone acetate found the specificity for the ICD codes alone to be <50% (9, 47). Therefore, we used stringent inclusion criteria to identify subjects with AAD, resulting in the exclusion of a small minority of patients with AAD not using mineralocorticoids.

When interpreting the results of the present study, it should be remembered that, by design, events before the start of the PDR in 2005 could not be examined. Only patients alive in 2005 were included. This might have caused us to underestimate the CVD risks, because the mortality in patients with AAD might be highest in the first year after diagnosis (3). However, restricting follow-up to recent years might not have been a disadvantage, because modern guidelines on AAD have generally emphasized the use of lower glucocorticoid doses than previously (48). Thus, studying data from 2005 onwards may have provided a more relevant risk assessment. The lack of data on obesity and smoking was a shortcoming. However, with previous studies of AAD reporting a low prevalence of both of these risk factors, positive confounding was less likely. Confounding by indication should also be considered. If higher doses of hydrocortisone were used to alleviate the symptoms of underlying illness linked to CVD, this would have affected the results. However, even in the highest tertile, the doses were generally too low to produce substantial anti-inflammatory effects. AAD is rare, and a stratified analysis of the potential dose effects will inevitably produce small cohorts with few events. This should be remembered when interpreting the results. Finally, despite correcting or adjusting for multiple factors, we could not exclude the effects of residual confounding.

In conclusion, using a large, population-based cohort, we have shown that the excess CVD mortality previously reported in patients with AAD results from the increased incidence and increased case-fatality rate in IHD. We found that most of this risk is carried by women. Moreover, we have confirmed the association between the glucocorticoid replacement doses and CVD previously observed with other forms of adrenal insufficiency. We have also shown that the mineralocorticoid replacement doses correlated with the incidence of CVD. We believe the added data from the present study have shown that the prescription of the lowest glucocorticoid and mineralocorticoid doses sufficient for well-being should be recommended and that closer monitoring of risk factors for CVD, especially in women, is required.

## Abbreviations:

ATC: Anatomical Therapeutical Chemical
aHR: adjusted hazard ratio
COPD: chronic obstructive pulmonary disease
CVD: cardiovascular disease
CeVD: cerebrovascular disease
HR: hazard ratio
ICD: International Statistical Classification of Diseases and Related Health Problems
ICD-10: 10th revision of International Statistical Classification of Diseases and Related Health Problems
IHD: ischemic heart disease
NPR: National Patient Register
PDR: Prescribed Drug Register
PY: person-years
